# Relationship between left ventricular shape and cardiovascular risk factors: comparison between the Multi-Ethnic Study of Atherosclerosis and UK Biobank

**DOI:** 10.1136/heartjnl-2024-324658

**Published:** 2025-01-16

**Authors:** Avan Suinesiaputra, Kathleen Gilbert, Charlene Mauger, David A Bluemke, Colin O Wu, Nay Aung, Stefan Neubauer, Stefan K Piechnik, Steffen E Petersen, Joao A C Lima, Bharath Ambale Venkatesh, Alistair Young

**Affiliations:** 1 Biomedical Engineering & Imaging Sciences, Faculty of Life Sciences & Medicine, King's College London, London, UK; 2 Anatomy and Medical Imaging, Faculty of Medical and Health Sciences, The University of Auckland, Auckland, New Zealand; 3 Mackie Research and Consulting, Auckland, New Zealand; 4 Radiology, School of Medicine and Public Health, University of Wisconsin-Madison, Madison, Wisconsin, USA; 5 Division of Intramural Research, National Heart, Lung, and Blood Institute, National Institutes of Health, Bethesda, Maryland, USA; 6 William Harvey Research Institute, NIHR Barts Biomedical Research Centre, Queen Mary University of London, London, UK; 7 Barts Heart Centre, St Bartholomew's Hospital, Barts Health NHS Trust, London, UK; 8 Oxford NIHR Biomedical Research Centre, Division of Cardiovascular Medicine, Radcliffe Department of Medicine, University of Oxford, Oxford, UK; 9 William Harvey Research Institute, Queen Mary University of London, London, UK; 10 Cardiology Division of the Departments of Medicine, Johns Hopkins Hospital, Baltimore, Maryland, USA; 11 Department of Cardiology, Johns Hopkins Bayview Medical Center, Baltimore, Maryland, USA

**Keywords:** Risk Factors, Cohort Studies, Magnetic Resonance Imaging

## Abstract

**Background:**

Statistical shape atlases have been used in large-cohort studies to investigate relationships between heart shape and risk factors. The generalisability of these relationships between cohorts is unknown. The aims of this study were to compare left ventricular (LV) shapes in patients with differing cardiovascular risk factor profiles from two cohorts and to investigate whether LV shape scores generated with respect to a reference cohort can be directly used to study shape differences in another cohort.

**Methods:**

Two cardiac MRI cohorts were included: 2106 participants (median age: 65 years, 54% women) from the Multi-Ethnic Study of Atherosclerosis (MESA) and 2960 participants (median age: 64 years, 52% women) from the UK Biobank (UKB) study. LV shape atlases were constructed from 3D LV models derived from expert-drawn contours from separate core labs. Atlases were considered generalisable for a risk factor if the area under the receiver operating characteristic curves (AUC) were not significantly different (p>0.05) between internal (within-cohort) and external (cross-cohort) cases.

**Results:**

LV mass and volume indices were differed significantly between cohorts, even in age-matched and sex-matched cases without risk factors, partly reflecting different core lab analysis protocols. For the UKB atlas, internal and external discriminative performance were not significantly different for hypertension (AUC: 0.77 vs 0.76, p=0.37), diabetes (AUC: 0.79 vs 0.77, p=0.48), hypercholesterolaemia (AUC: 0.76 vs 0.79, p=0.38) and smoking (AUC: 0.69 vs 0.67, p=0.18). For the MESA atlas, diabetes (AUC: 0.79 vs 0.74, p=0.09) and hypercholesterolaemia (AUC: 0.75 vs 0.70, p=0.10) were not significantly different. Both atlases showed significant differences for obesity.

**Conclusions:**

The MESA and UKB atlases demonstrated good generalisability for diabetes and hypercholesterolaemia, without requiring corrections for differences in mass and volume. Significant differences in obesity may be due to different relationships between obesity and heart shapes between cohorts.

**WHAT IS ALREADY KNOWN ON THIS TOPIC**

- Common risk factors, such as hypertension and obesity, have been linked with changes in the morphology and function of the heart. Atlas-based individual heart shape scores derived from one cohort can be used to quantify heart health relative to the same cohort. It is not known how atlas-based shape scores generalise to an external cohort. A direct comparison of such a metric between cohorts has not been performed.

**WHAT THIS STUDY ADDS**

- Despite differences in volumes and masses, atlas-based shape scores derived from one cohort can be applied directly to the other cohort and vice versa, without significant differences in the discriminative performance, for many risk factors including diabetes and hypercholesterolaemia. The atlas-based shape scores are not interchangeable for obesity, which suggests different heart morphological alterations between the two cohorts due to obesity.

**HOW THIS STUDY MIGHT AFFECT RESEARCH, PRACTICE OR POLICY**

- This study provides new insights of the cross-cohort relationships between left ventricular shapes with cardiovascular risk factors. Some risk factors can be used interchangeably, while others need further investigation. Generally, care must be taken to report the generalisability of a machine learning model derived exclusively from a single cohort to a separate cohort.

## Introduction

Large-scale cardiac studies have proven invaluable for the investigation of risk factors associated with cardiovascular disease (CVD). Established risk factors such as hypertension and obesity have been linked with heart shape changes.^1^ Left ventricular (LV) shape atlases have been used to find associations between geometrical features of the heart and future adverse events.^2^

Atlas-based shape scores can facilitate a deeper understanding of the pathogenetic mechanisms underlying processes associated with CVD.^3^ Early identification of subclinical CVD phenotypes can be derived from an LV shape atlas to get morphological features that are most correlated with a CVD risk factor or outcome. Shape scores can be used as phenotypic markers relative to a healthy reference cohort to determine high risk groups, evaluate disease progression and treatment and facilitate optimal treatment plans. Previous studies^4–6^ have investigated associations between atlas-based LV shape scores with CVD risk factors in a single cohort, but a direct comparison of atlas-based metrics between cohorts has not been performed.

Comparisons between cohorts are difficult, due in part to potential differences in image analysis protocols. Variations in image analysis may give rise to bias in measured ventricular mass and volumes,^7^ masking differences in risk factor exposure, lifestyle and demographics. However, if the shape measures are robust, the associations between LV shapes and CVD risk factors should be able to be replicated between cohorts. In this study, we compared two statistical shape atlases constructed from Multi-Ethnic Study of Atherosclerosis (MESA) and the UK Biobank (UKB) cohorts independently. We investigated whether relationships between LV shapes and differing CVD risk profiles are similar between cohorts and whether the shape scores derived from one cohort can be applied to the other.

## Methods

### Cohort characteristics

MESA was a population-based observational study of 6814 participants in the USA between 2000 and 2002,^8^ free of CVD at enrolment, where 3015 of them returned for the follow-up MRI examinations between 2010 and 2012 (median time: 9.4 years).^9^ The UKB imaging study is a prospective cohort, aiming to scan 100 000 participants from 22 centres across the UK.^10^ The first phase of 5065 UKB cardiovascular magnetic resonance (CMR) examinations between 2014 and 2015 were manually analysed^11^ to establish the reference values for cardiac function. In this study, we included the MESA follow-up and the first phase UKB CMR examinations because of the manual analysis availability and the same imaging protocol, thus avoiding bias due to different MRI parameters.

Both studies comprised steady-state free precession cine images acquired using 1.5 Tesla scanners (MESA: Signa LX, GE Healthcare, and Avanto or Espree, Siemens Healthcare, and UKB: MAGNETOM Aera, Syngo Platform VD13A, Siemens Healthcare). CMR studies without core lab myocardial contours were excluded. Cases with history of cardiovascular events, revascularisation procedures and historical cardiac-related diseases were also excluded (see online supplemental table 1 for excluded events, disease and interventions).

10.1136/heartjnl-2024-324658.supp1 Supplementary data

**Figure 1:**
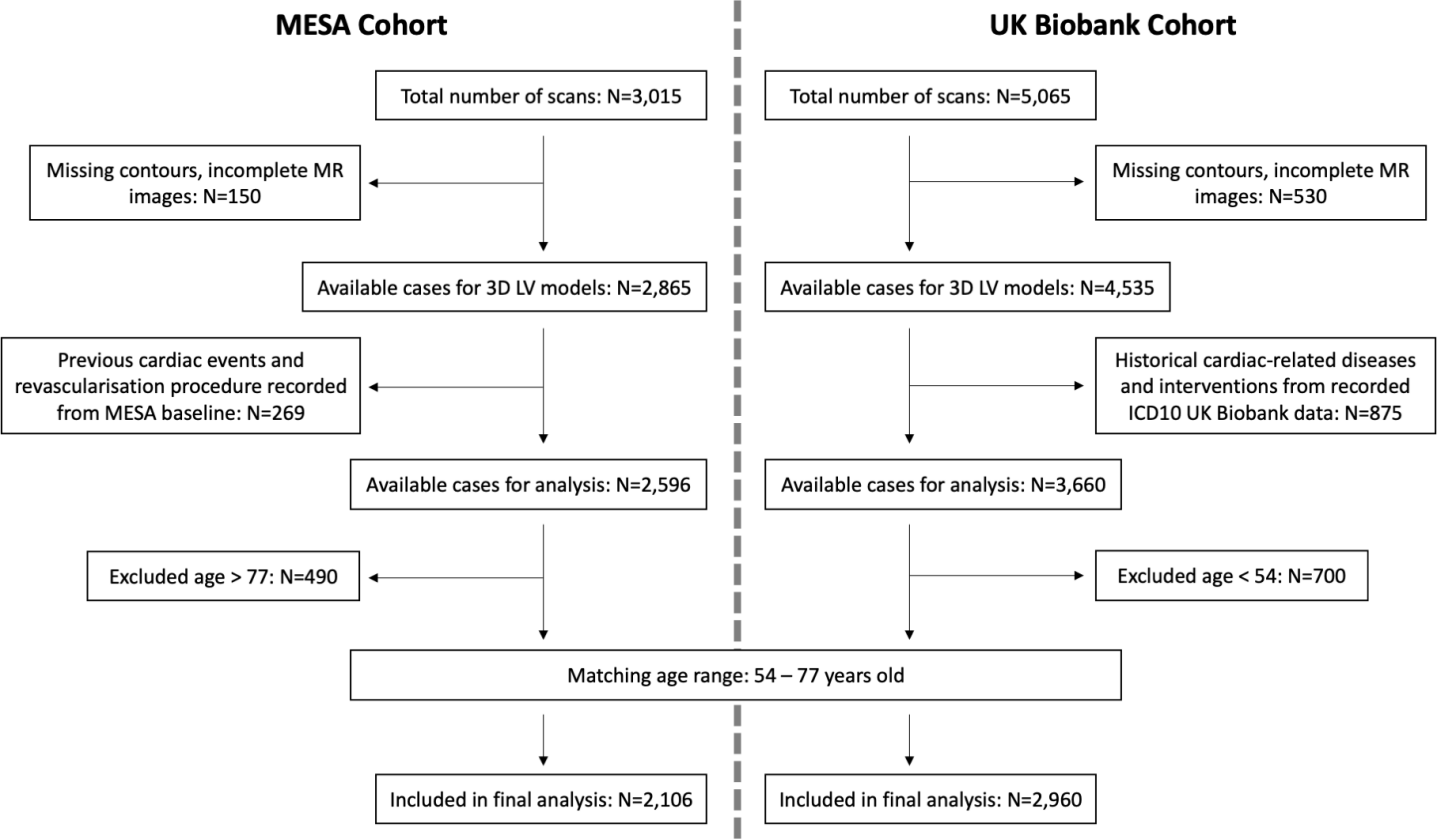
Inclusion and exclusion criteria for the Multi-Ethnic Study of Atherosclerosis (MESA) and UK Biobank cohorts. LV, left ventricular.

The remaining cases available were 2596 from MESA (54% women, median age: 69 years, range: 54–94) and 3660 from UKB (53% women, median age: 62 years, range: 44–77). The proportion of sex was matched between the two cohorts, but not the age range. MESA participants were generally older. To remove bias due to age differences, we only included cases from the same age range, that is, 54–77 years old, which resulted in 2106 MESA cases (median age: 65 years, 54% women) and 2960 UKB cases (median age: 64 years, 52% women). A full inclusion process diagram is shown in figure 1.

### Cardiovascular risk factors

We follow standard guidelines to determine CVD risk factors. Hypertension was defined for participants who reported taking antihypertensive medication, or if either systolic blood pressure (SBP)≥140 mm Hg or diastolic blood pressure (DBP)≥90 mm Hg. If an antihypertensive medication was used, SBP was increased by a constant 15 mm Hg and DBP by 10 mm Hg.^12^ The SBP and DBP thresholds were reduced to 130 and 80 mm Hg, respectively, if a participant had diabetes.^13^ Diabetes was determined with glycated haemoglobin (HbA1c) biomarkers above 48 mmol/mol or 6.5%,^14^ had taken insulin medication or had a known diagnosis of diabetes. Hypercholesterolaemia was determined by the primary severe hypercholesterolaemia (low-density lipoprotein cholesterol (LDL-C)≥190 mg/dL) and very high risk for future atherosclerotic cardiovascular disease events groups (LDL-C≥100 mg/dL, age≥65 years and lipid-lowering medication) according to the American College of Cardiology/American Heart Association 2018 Guideline.^15^ Body mass index (BMI) above 30 kg/m^2^ was used to determine obesity.^16^ Smoking was determined for current and previous smokers. All participants without any risk factors were identified as the no-risk group.

### Image analysis

**Figure 2:**
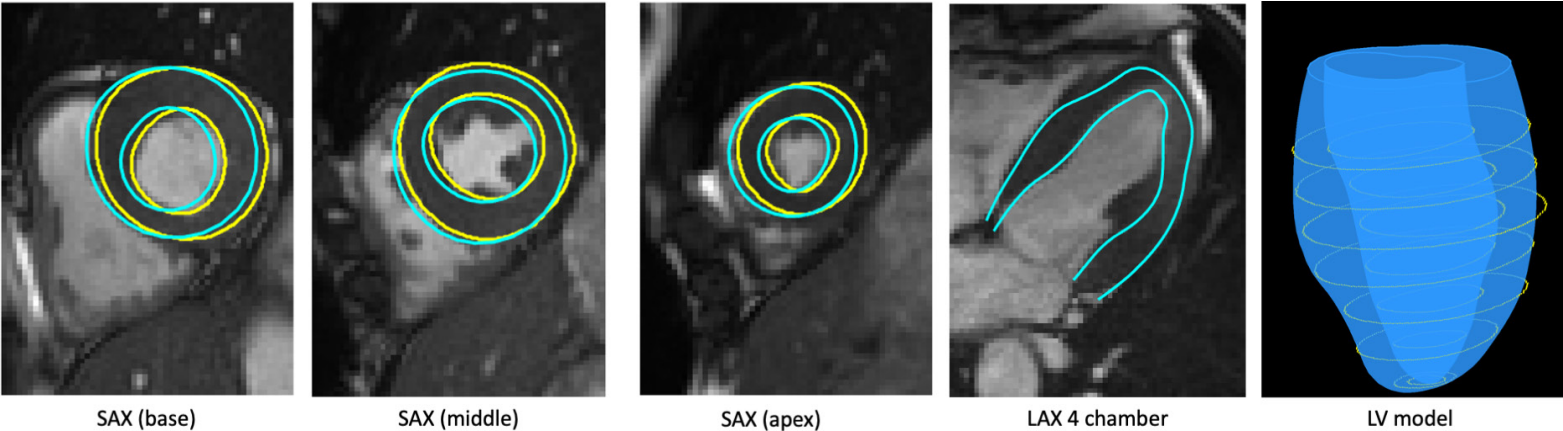
LV model customisation example to a cardiovascular magnetic resonance study. Yellow contours are expert-drawn contours. After the automated pipeline processing to fit the LV model with the manual contours, a 3D LV model was generated. Blue contours are the intersection of fitted LV model with the images. Note that manual contours were only available on short-axis views. The long-axis four chamber view is shown to demonstrate the correctness of the fitted model. LAX, long axis view; LV, left ventricular; SAX, short axis view.

For MESA, core lab readers (Johns Hopkins Medical Centre, Baltimore, Maryland, USA) analysed images using Cardiac Image Modeller software (V.6.2, Auckland MR Research Group, University of Auckland, New Zealand).^17^ For UKB, core lab readers (Queen Mary University, London, UK and Oxford University, UK) analysed images using cvi^42^ postprocessing software (V.5.1.1, Circle Cardiovascular Imaging, Calgary, Alberta, Canada).^11^ For both studies, the end-diastolic (ED) frame was selected as the first frame of the series and the end-systolic (ES) frame was selected as the smallest blood pool area in the mid-ventricular slice. Papillary muscles were included in the blood pool. The most basal slices were determined by at least 50% of the blood pool surrounded by the myocardium. A finite element LV shape model was automatically customised to fit to manual contours^18^ (see figure 2). Standard ED volume, ES volume, mass and ejection fraction were derived from the LV model using numerical integration (mass being myocardial volume multiplied by 1.05 g/mL). Indexed volumes and mass were calculated by dividing the measurement by body surface area.

### LV atlases

Two separate atlases were created, one for each cohort. Homologous surface sample points from the endocardium and epicardium at ED and ES were concatenated as a single vector of $\left[x_{1},y_{1},z_{1},\ldots ,\;x_{N},\;y_{N},\;z_{N}\right]$ , where N is the total number of sample points. Shape variations due to translation and rotation were removed using generalised Procrustes alignment. Scaling was kept intact, that is, not removed by the alignment. Principal component analysis (PCA) was then applied to the differences from the mean shape.

### Relationships between shape and risk factors

We compared LV shapes with differing CVD risk profiles between the two cohorts by quantifying the discriminative performance of a linear model trained to predict the presence of a risk factor based on the LV shape. Partial least squares regression (PLSR) was used to train predictive models for each risk factor separately by using cases with that risk factor as a *risk group* and cases without any risk factor as a *no-risk group*. Age, sex (0=female, 1=male) and PCA shape scores were included as predictor variables. The number of shape scores included was determined by the number of principal components that explained 99.9% of the total variance. The optimal number of PLSR components was estimated by using fivefold cross-validation.

Leave-one-out cross-validation performance was aggregated over the test cases using the previously estimated number of PLSR components. For the external cohort discriminative performance, all internal cohort cases were used to train a PLSR model. The external cohort LV shapes were first aligned to the mean shape of the internal LV atlas, followed by subtraction by the mean shape. External shape scores were subsequently obtained by computing the dot product of the aligned shape vectors with the PCA component vectors.

### Statistical analysis

Areas under the receiver operating characteristics (ROC) curves (AUC) were used as a measure of the strengths of relationships between shape scores and risk factors. CIs around AUC values were calculated by using the bootstrapping method. All analyses were performed using the R statistical tool (V.4.3.2, R Core Team, 2023). The PLSR models were built using the caret package^19^ and the AUC analysis was performed by using pROC package.^20^ Differences between internal and external performance were tested using the unpaired t-test with unequal sample size and unequal variance. For the no-risk group comparison between cohorts, propensity score was performed to match the exact age and sex cases and each pair was selected by using the nearest matching method. For all tests, significance was declared at p<0.05.

## Results

### Cohort characteristics

MESA cases overall had higher BMI and HbA1c compared with UKB (table 1), which correlated with significantly higher prevalence of obesity and diabetes. The prevalence of hypercholesterolaemia was significantly higher in UKB, while there were no significant differences in the prevalence of hypertension and smoking. SBP and DBP on average were lower in MESA. MESA participants had higher lipid-lowering medication use. Table 1 also shows 30% bigger mass in MESA, but 22–33% smaller in volumes.

**Table 1:** Cohort characteristics

Cohort	MESA, n=2106	UK Biobank, n=2960	P value
Female	1138 (54%)	1534 (52%)	0.12
Age	65 (54, 77)	64 (54, 77)	<0.001
BMI (kg/m^2^)	28.3±5.2	26.6±4.3	<0.001
SBP (mm Hg)	121±19	141±19	<0.001
DBP (mm Hg)	69±10	82±12	<0.001
HbA1c (mmol/mol)	40.9±9.1	35.7±4.9	<0.001
HDL cholesterol (mg/dL)	56±17	58±15	<0.001
LDL cholesterol (mg/dL)	109±32	136±37	<0.001
Triglycerides (mg/dL)	111±62	148±82	<0.001
Total cholesterol (mg/dL)	187±36	224±42	<0.001
Antihypertensive medication	978 (46%)	720 (24%)	<0.001
Insulin medication	50 (2.4%)	22 (0.7%)	<0.001
Lipid-lowering medication	655 (31%)	678 (23%)	<0.001
Diabetes	373 (18%)	159 (5.4%)	<0.001
Obesity	676 (32%)	532 (18%)	<0.001
Hypertension	1147 (54%)	1634 (55%)	0.6
Hypercholesterolaemia	145 (6.9%)	439 (15%)	<0.001
Smoking	891 (42%)	1182 (40%)	0.09
No risk	397 (19%)	645 (22%)	0.011
LVEDVI (mL/m^2^)	64±13	78±14	<0.001
LVESVI (mL/m^2^)	24±8	32±9	<0.001
LVMI (g/m^2^)	65±13	50±10	<0.001
LVEF (%)	63±7	59±6	<0.001

Categorical variables are represented as ‘size (percentage)’, while numerical variables are shown as ‘mean (SD) (minimum, maximum)’.

Statistical tests were performed between MESA and UK Biobank cohorts with Wilcoxon rank sum test for continuous variables and Pearson’s χ^2^ test for categorical variables.

BMI, body mass index; DBP, diastolic blood pressure; HbA1c, glycated haemoglobin; HDL, high-density lipoprotein; LDL, low-density lipoprotein; LVEDVI, left ventricular end diastolic volume index; LVEF, left ventricular ejection fraction; LVESVI, left ventricular end systolic volume index; LVMI, left ventricular mass index; MESA, Multi-Ethnic Study of Atherosclerosis; SBP, systolic blood pressure.

A total of 397 (19%) and 645 (22%) cases without any cardiovascular risk factors were identified from MESA and UKB, respectively. After full propensity score matching, 363 no-risk factor cases were compared for LV volumes and mass indices in table 2. Indexed LV end-diastolic volume (LVEDVI), indexed LV end-systolic volume (LVESVI), indexed LV mass (LVMI) and LV ejection fraction (LVEF) were still significantly different. LVEDVI and LVESVI were higher in the UKB cohort, with lower LVMI and LVEF.

**Table 2:** Comparison of LV volumes and mass for the no-risk groups after full age and sex propensity score matching

	MESA, n=363	UK Biobank, n=363	P value
Age	61.0 (54.0, 76.0)	61.0 (54.0, 76.0)	>0.9
Female	194 (53%)	194 (53%)	>0.9
LVEDVI (mL/m^2^)	66±12	79±14	<0.001
LVESVI (mL/m^2^)	24±6	32±8	<0.001
LVMI (g/m^2^)	62±11	48±9	<0.001
LVEF (%)	63±6	59±6	<0.001

All values are shown as ‘mean (SD) (minimum, maximum)’.

Statistical tests were performed between MESA and UK Biobank cohorts with Wilcoxon rank sum test for continuous variables and Pearson’s χ^2^ test for categorical variables.

LV, left ventricular; LVEDVI, left ventricular end-diastolic volume indexed; LVEF, left ventricular ejection fraction; LVESVI, left ventricular end-systolic volume indexed; LVMI, left ventricular mass index; MESA, Multi-Ethnic Study of Atherosclerosis.

### LV shape atlases

**Figure 3:**
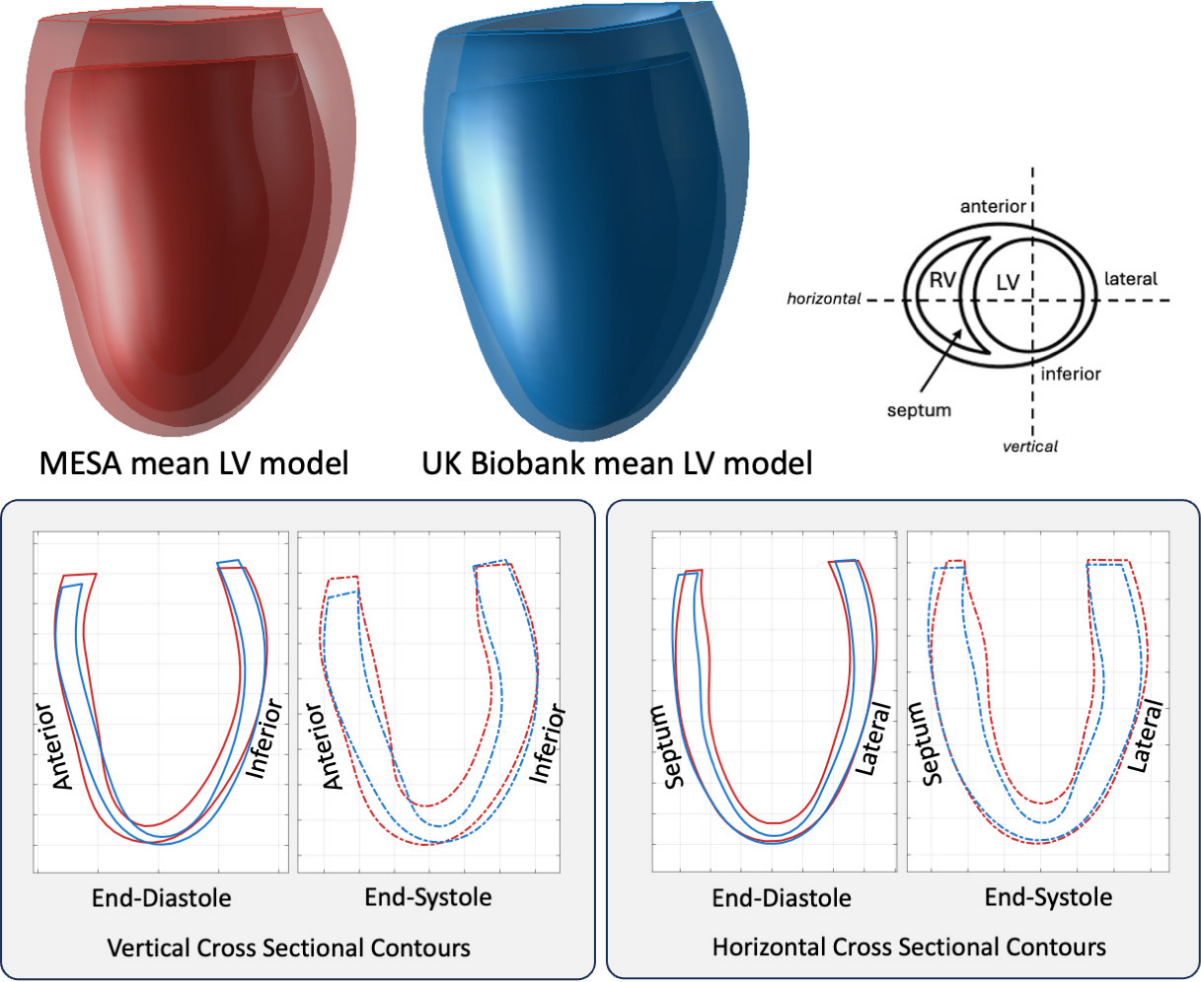
Top: Mean LV models from MESA (red) and UK Biobank (blue) atlases, where each subfigure shows end-diastole (bigger) and end-systole (smaller) LV shapes. Bottom: Horizontal and vertical cross-sectional contours, where solid lines are end-diastole and dashed lines are end-systole contours. LV, left ventricle; MESA, Multi-Ethnic Study of Atherosclerosis; RV, right ventricle.

Figure 3 shows the mean LV models of MESA and UKB cohorts after the Procrustes shape alignment. There were noticeable differences between MESA and UKB shapes as shown by the cross-sectional contours. The mean UKB LV shape was thinner around the apical region than MESA, consistent with smaller mass. The endocardial contours that define the cavity volume in MESA were smaller than UKB both at ED and ES frames, consistent with smaller LVEDVI and LVESVI values. The endocardial contours of the mean UKB LV shapes have more curvature particularly around the apical septum and lateral wall.

After PCA, the number of principal components needed to explain 99.9% of total shape variance was 176 and 210 for the MESA and UKB atlases, respectively. These have resulted in 98.1% and 97.8% dimensionality reductions for MESA and UKB atlases from the total original LV shape dimension of 9420 points. The first five principal components of both atlases depicted similar modes of shape variations, although not in the same order (see the animations in online supplemental figure 1).

10.1136/heartjnl-2024-324658.supp3 Supplementary video

### Atlas-based shape scores

The optimal number of PLSR components was eight from the MESA cohort for all risk factors and varied between 6 and 23 for the UKB cohort. This suggested more variations in the principal components of the UKB atlas compared with MESA for the PLSR to regress to each risk factor.

**Figure 4:**
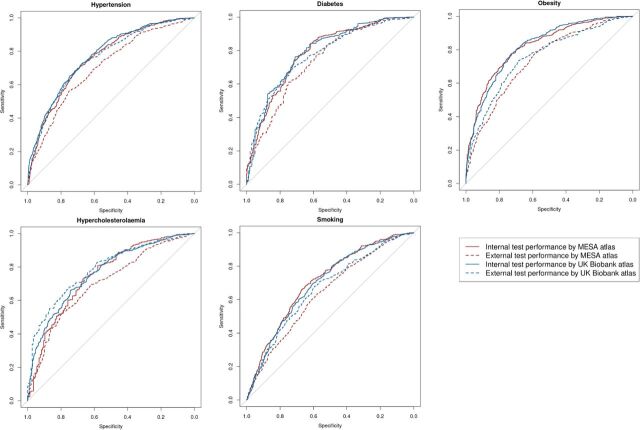
Receiver operating characteristics curves of shape-based atlas discrimination (Multi-Ethnic Study of Atherosclerosis (MESA) and UK Biobank) on five cardiovascular risk factors: hypertension, diabetes, obesity, hypercholesterolaemia and smoking. Each figure contains internal (solid line) and external (dashed line) test cases for both shape-based atlas score discriminations.

ROC curves from the shape-based atlases are shown in figure 4, where each figure shows internal and external curves from the same atlas. As shown in table 3, AUCs ranged between 0.64 (MESA external smoking) and 0.82 (both MESA and UKB internal obesity). Internal test case performance was similar between MESA and UKB (full overlap of AUC confidence intervals and all p>0.05). The PLSR model prediction summary with the optimal threshold based on the maximum balance between sensitivity and specificity is given in online supplemental table 2.

10.1136/heartjnl-2024-324658.supp2 Supplementary data

**Table 3:** Comparisons of model prediction performance between internal and external test cohorts

	Internal AUC	External AUC	P value
Hypertension			
MESA atlas	0.76 (0.73, 0.78)	0.70 (0.68, 0.73)	p=0.003
UKB atlas	0.77 (0.75, 0.79)	0.76 (0.73, 0.78)	p=0.37
Diabetes			
MESA atlas	0.79 (0.75, 0.82)	0.74 (0.69, 0.78)	p=0.09
UKB atlas	0.79 (0.75, 0.83)	0.77 (0.74, 0.81)	p=0.48
Obesity			
MESA atlas	0.82 (0.80, 0.85)	0.74 (0.71, 0.77)	p<1e-05
UKB atlas	0.82 (0.80, 0.85)	0.75 (0.72, 0.78)	p=0.0006
Hypercholesterolaemia			
MESA atlas	0.75 (0.70, 0.80)	0.70 (0.67, 0.73)	p=0.10
UKB atlas	0.76 (0.73, 0.79)	0.79 (0.74, 0.83)	p=0.38
Smoking			
MESA atlas	0.71 (0.68, 0.74)	0.64 (0.61, 0.67)	p=0.0008
UKB atlas	0.69 (0.67, 0.72)	0.67 (0.63, 0.70)	p=0.18

Values are the area under the receiver operating characteristic curves (AUC) with 95% CI. The external test cases for the MESA atlas were UKB shapes projected to the MESA atlas, while the external test cases for the UKB atlas were MESA shapes projected to the UKB atlas. Statistical tests were performed between internal and external AUC values with unpaired receiver operating characteristics test.

MESA, Multi-Ethnic Study of Atherosclerosis; UKB, UK Biobank.

Of 10 discriminatory ability comparisons in table 3, six models maintained their performance between internal and external test cases, indicated by the AUC overlap between 95% CI values and non-significant p values. Visually, external versus internal ROC curves of these models were similar (figure 4). The UKB atlas was generalisable in more risk factors (hypertension, diabetes, hypercholesterolaemia and smoking) than the MESA atlas (diabetes and hypercholesterolaemia). For diabetes and hypercholesterolaemia, the two atlases were interchangeable. For obesity, although the MESA and UKB atlases had similar internal test performance (both AUC=0.82 with 100% CI overlaps: 0.75–0.82), both atlases had a significant drop in external test performance (p<0.05).

## Discussion

LV mass and volume calculations were significantly different between MESA and UKB (table 1), even after matching age and sex in the no-risk group (table 2). These differences may be due to different average blood pressure and cholesterol levels (although within the normal range), but part is likely due to contouring protocol differences between the core labs. Systematic differences in LV mass and volume between core labs have been documented previously.^21^ These differences can introduce potential biases related to the cross-sectional nature of the two cohorts. In this study, we did not attempt to correct for biases between core lab analyses but rather investigated whether atlas-based shape scores could maintain discriminatory ability to identify LV shapes with risk factors across cohorts.

**Figure 5:**
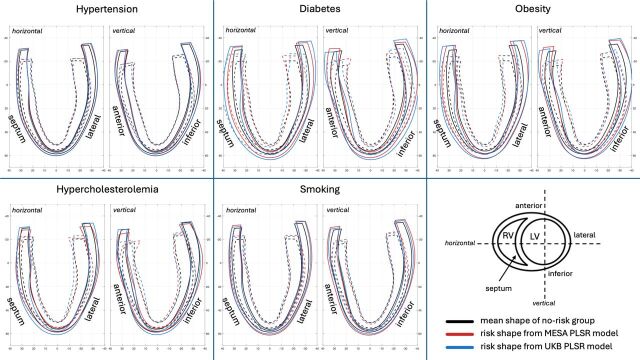
LV shape comparisons of the most associated risk factors from both cohorts (red lines are contours from Multi-Ethnic Study of Atherosclerosis (MESA) partial least squares regression (PLSR) models and blue lines are contours from UKB PLSR models). The mean LV shape contours estimated from the no risk group are shown by the black lines. Solid lines are end-diastolic contours and dashed lines are end-systolic contours. Two cross-sectional contours are shown from horizontal direction (left panel) showing septal and lateral walls, and vertical direction (right panel) showing anterior and inferior wall. The cross-sectional diagram is also shown at the bottom right of the figure. LV, left ventricle; RV, right ventricle.

Within each cohort, AUC values from internal test cases were similar (all had CI overlaps and p>0.05), which corroborated previous cohort-specific studies. In the MESA cohort, Medrano-Gracia *et al* 4 demonstrated that LV shapes were associated with smoking, hypertension and diabetes risk factors, which was further explored by Mauger *et al* 2 to identify high-risk groups of developing heart diseases within 10 years. In the UKB cohort, Petersen *et al* 22 show associations of modifiable risk factors, including hypertension, smoking, high cholesterol and diabetes, with all four chambers of the heart. The cross-sectional contours associated with each risk factor shown in figure 5 further highlight similar morphological shape effects between MESA and UKB PLSR models.

For diabetes and hypercholesterolaemia, there were no significant differences found between internal and external cases discriminated by both atlases. For obesity, internal and external AUC values from both cohorts were significantly different (table 3). The differences in obesity discriminative ability may be indicative of different shape–obesity relationships operating in the two cohorts. Several studies have found a complex relationship between cardiac shape and obesity, including non-linear positive associations^23^ and non-homogenous distribution of regional wall thickness in an obese group.^24^

In this study, we followed the current standard guideline to define obesity by using a BMI threshold.^16^ However, BMI only accounts for height and weight, which ignores factors that include body fat, age, sex and ethnicity.^25^ Women tend to have more body fat than men at the same BMI and older adults tend to have more fat and less muscle.^26^ Different ethnic groups may have different cut-off values for BMI.^27^ Nevertheless, this study has shed light on differences in the shape-based atlas scores to discriminate LV shapes with obesity across cohorts, suggesting the need for ethnic-specific based measure for obesity beyond BMI to improve risk stratification and patient management.

### Clinical importance

This study has demonstrated the generalisability of shape-based atlas scores to associate patients at risk for CVD outside the training dataset. An LV model can be quantified directly from CMR images^18^ then projected onto the atlas to get patient-specific scores. These scores contain additional diagnostic measures related to the patient heart function and morphology to stratify patients according to CVD risk factors from the reference population. A more elaborate report from the CMR examination can be generated, allowing richer diagnostic information in clinical practice.

### Limitations

Ethnicity has been known as a confounding factor in the development of heart failure. In this study, we did not adjust the PLSR models by ethnicity because the difference of ethnicity distributions between MESA and UKB could not be corrected. The ethnic groups in MESA are more widespread among black African American (25%), Chinese American (13%), Hispanic (20%) and white Caucasian (42%), while the UKB population is more concentrated on white British/Irish (94.4%).

The effect of multiple risk factors was not considered due to lack of cases for participants with different combinations of risk factors. More detailed analyses with a larger number of cases with multiple risk factors are needed to evaluate the interactions between LV shape and multifactorial risk factors.

## Conclusion

Both MESA and UKB atlases had similar internal (within-cohort) performance to associate LV shapes with differing risk factor profiles. Despite differences in volumes and masses, atlas-based LV shape scores for diabetes and hypercholesterolaemia generalised into external cohorts without significant differences. However, the atlases were not interchangeable for obesity.

## Data Availability

Data may be obtained from a third party and are not publicly available. The MESA CMR images and their clinical and demographic data used in this study are available on request to the MESA Coordinating Centre at[https://www.mesa-nhlbi.org]. The UK Biobank CMR images and their clinical and demographic data used in this study are available on request to the UK Biobank at [https://www.ukbiobank.ac.uk]. The principal components of both MESA and UK Biobank derived in this study to build the PLSR model are available from the Cardiac Atlas Project website [https://www.cardiacatlas.org]. Source codes are available from [https://cardiacatlasproject.github.io/MESA-UKB-LVAtlas/]
